# Development of a CHD risk prediction model using novel lipid markers based on lipidomics and VAP technology

**DOI:** 10.3389/fmed.2026.1926464

**Published:** 2026-08-26

**Authors:** Xiehui Chen, Lvwen Ning, Zixi Chen, Qidi Ying, Wei Hu, Yanwen Qin, Ahlam Mohamed Yusuf, Yinling Wang, Ying Wang, Zhanke Wang

**Affiliations:** 1 Department of Geriatrics, Shenzhen Longhua District Central Hospital, Shenzhen, China; 2 Ningbo-Marshall International Joint Medical Laboratory, Centre for Medical Research, Ningbo No. 2 Hospital, Ningbo, China; 3 Ningbo Key Laboratory for Precision Detection of Lipoprotein Subfractions by VAP, Ningbo Meikang Shengde Medical Laboratory, Ningbo, China; 4 Department of Cardiology, Beijing Anzhen Hospital, Capital Medical University, Beijing, China; 5 Department of Cardiology, The First Affiliated Hospital of Ningbo University, Ningbo, China; 6 Department of Clinical Laboratory, Ningbo Yinzhou Hospital of Traditional Chinese Medicine, Ningbo, China; 7 Department of Clinical Laboratory, Hangzhou First People’s Hospital, Hangzhou, China

**Keywords:** coronary heart disease, differential lipids, lipidomics, risk prediction model, ROC curve, vertical auto profile

## Abstract

**Objective:**

This study aimed to develop a novel risk prediction model for coronary heart disease (CHD) based on lipid subfraction and lipidomics data.

**Methods:**

Lipid subfraction analysis was conducted on 624 subjects (312 CHD, 312 controls) using Vertical Auto Profile (VAP). The participants were randomly divided into a training cohort (*n* = 422, 211 CHD, 211 controls) and a validation cohort (*n* = 202, 101 CHD, 101 controls) at a 2:1 ratio, stratified by CHD status. Untargeted lipidomics was performed on 16 subjects (8 CHD, 8 controls) randomly selected from the total enrolled population using UHPLC-Q-TOF/MS. Lasso regression with ten-fold cross-validation was applied to screen key predictive factors, followed by multivariate logistic regression to construct a risk prediction model. The false discovery rate (FDR) was controlled using the Benjamini–Hochberg procedure for multiple comparisons. Model discriminative performance was evaluated using ROC curve analysis.

**Results:**

A total of 2,335 lipid molecules across eight classes were identified. After FDR correction, the differential lipids remained statistically significant. CHD patients exhibited significant upregulation of RLP-C and sdLDL-C-related lipid components, while HDL2B-related lipids were markedly downregulated. KEGG pathway enrichment analysis revealed that differential lipids were mainly enriched in glycerophospholipid metabolism, choline metabolism in cancer, and fat digestion and absorption. Multivariate logistic regression identified Lp(a) (OR = 1.288), RLP-C (OR = 3.848), sdLDL-C (OR = 5.317), age (OR = 1.053), systolic blood pressure (OR = 1.075), and fasting plasma glucose (OR = 3.903) as independent risk factors for CHD, whereas HDL2B (OR = 1.416 × 10^−6^) was a protective factor. In the validation cohort, the model achieved an AUC of 0.867 (95% CI: 0.8087–0.9252). Using an optimal cutoff of 0.4726 (Youden index = 0.628), the model demonstrated a sensitivity of 81.4% and a specificity of 81.4%.

**Conclusion:**

This study successfully constructed a multivariate logistic regression risk prediction model integrating novel lipid subfractions (sdLDL-C, RLP-C, HDL2B, Lp(a)) and routine clinical variables (age, SBP, FPG). The model exhibits acceptable predictive performance and may provide a complementary tool for CHD risk assessment, pending external validation in larger prospective cohorts.

## 1 Introduction

Coronary heart disease (CHD) is defined as myocardial dysfunction and organic lesions caused by coronary artery stenosis and inadequate blood supply. It has surpassed cancer as the leading cause of death worldwide, accounting for approximately 17.9 million deaths annually, a figure that continues to rise with the aging population and the increasing prevalence of metabolic diseases (1–3). However, the pathological mechanisms of CHD are complex, resulting from the interplay of genetic, environmental, and metabolic disturbances that lead to vascular endothelial injury, lipid deposition, and the cascade activation of inflammatory responses (4, 5). Recent advances in metabolomics have further elucidated the pivotal role of lipid species in the initiation and progression of atherosclerotic plaques (6, 7). Additionally, large-scale prospective studies have confirmed that subtle alterations in circulating lipids precede the clinical onset of CHD by several years (8, 9).

The conventional lipid profiles (total cholesterol [TC], triglycerides [TG], low-density lipoprotein cholesterol [LDL-C], and high-density lipoprotein cholesterol [HDL-C]) and myocardial injury markers are widely used in the clinical diagnosis and treatment of CHD (10–12). However, these indicators suffer from substantial limitations, which include insufficient specificity, limited sensitivity for early diagnosis, and an inability to accurately reflect the precise pathological state of the disease. Consequently, some patients with occult or early-stage CHD miss the window for intervention for their conventional lipid parameters show no significant abnormalities (13). In particular, conventional LDL-C fails to capture the atherogenic potential of remnant cholesterol and small, dense LDL particles, which are now recognized as key drivers of residual cardiovascular risk (14, 15). Therefore, identifying novel biomarkers that are closely associated with the pathological progression of CHD and possess both high sensitivity and specificity is of great clinical significance for achieving early disease screening, risk stratification, and precision management.

In response to the shortcomings of traditional biomarkers, significant efforts have been made to develop more accurate risk prediction models. Based on the German Prospective Cardiovascular Munster (PROCAM) cohort study, Assmann et al. established a coronary heart disease (CHD) risk prediction model applicable to European populations that incorporates multiple cardiovascular risk factors (16). The model includes eight independent risk factors, including age, LDL-C, smoking, HDL-C, systolic blood pressure, family history of premature myocardial infarction, diabetes, and TG, which achieves an area under the curve (AUC) of 82.4% for CHD risk prediction. In another study, the curve of receiver operating characteristic (ROC) was used to evaluate the predictive efficacy of the LDL/HDL ratio for CHD. Multivariate logistic regression analysis identified LDL-C, HDL-C, and the LDL/HDL ratio as independent risk factors for CHD, with AUCs of 0.574, 0.625, and 0.668, respectively (17). In addition, A previous PROCAM study, involving 4,559 male participants aged 40–64 years over a 6-year follow-up, found that 186 subjects developed atherosclerotic CHD, including 134 non-fatal myocardial infarctions (MIs) and 52 CHD deaths. Univariate analysis demonstrated a significant inverse association between HDL-C levels and the incidence of atherosclerotic CHD (18). However, these models, while valuable, still rely predominantly on conventional lipid parameters, which are increasingly recognized as insufficient.

Conventional lipid parameters such as TC, TG, LDL-C, and HDL-C are relatively homogeneous in their predictive value for CHD and fail to reflect the critical heterogeneity of lipoproteins. A substantial number of CHD patients have normal or even low conventional LDL-C levels yet still experience plaque progression and myocardial infarction, a phenomenon known as the “residual risk.” This paradox arises because traditional assays measure cholesterol content but not the number, size, density, or functional properties of lipoprotein particles. In addition, LDL particles are highly heterogeneous. Small dense LDL (sdLDL) particles are more atherogenic than large, buoyant LDL due to their greater susceptibility to oxidation, higher affinity for arterial proteoglycans, and longer plasma residence time. Similarly, HDL particles vary in their cholesterol efflux capacity, with smaller HDL subfractions (e.g., HDL3) exhibiting different functionalities compared to larger ones (e.g., HDL2). Furthermore, TC, TG, and LDL-C are easily influenced by short-term diet, obesity, insulin resistance, glucose-lowering and lipid-lowering medications, and inflammatory status. Moreover, these indexes exhibit large fluctuations and poor stability, which may lead to significant errors in long-term risk prediction. Crucially, these conventional markers only reflect cholesterol content and cannot capture the core pathogenic mechanisms of lipoproteins, such as inflammation, oxidative modification, endothelial injury, and plaque deposition (19–22).

Recent technological advances, particularly VAP technology and advanced lipidomics, have enabled the precise quantification of lipoprotein subfractions and individual lipid species. The technology of VAP utilizes ultracentrifugation to directly measure cholesterol content within specific lipoprotein subfractions, including VLDL, IDL, LDL (further separated into LDL1-4), HDL (separated into HDL2 and HDL3, and further into subfractions like HDL2A, HDL2B, HDL2C, HDL3A, HDL3B, HDL3C), as well as Lipoprotein(a) [Lp(a)] and Remnant Lipoprotein Cholesterol (RLP-C). Meanwhile, untargeted and targeted lipidomics using UHPLC-Q-TOF/MS allows for the comprehensive identification and quantification of thousands of individual lipid molecules across different classes such as glycerophospholipids, sphingolipids, and glycerolipids, providing unprecedented molecular detail. Thus integrating these technologies offers a powerful approach to dissect the complex lipid dysregulation underlying CHD and to identify novel, more precise biomarkers.

In this study, we aimed to develop a novel CHD risk prediction model based on lipid subfraction data. The data could clarify the respective influence weights of different lipid subfractions on CHD occurrence, address the limitations of conventional lipid indicators in terms of specificity and accuracy for CHD risk assessment, and enhance the precision of CHD risk prediction. Specifically, this study utilizes a discovery-validation two-stage design. The untargeted lipidomics is firstly employed to screen for differential lipid molecules between CHD patients and healthy controls. Then the VAP technology is used to quantify lipid subfractions in a larger cohort, and Lasso regression and multivariate logistic regression are applied to construct a risk prediction model. The goal is to provide a basis for early clinical identification of high-risk CHD populations and the formulation of individualized preventive intervention strategies, thereby reducing CHD-related disability and mortality.

## 2 Materials and methods

### 2.1 Study participants

This study employed a discovery–validation two-stage design. The discovery cohort comprised 16 participants (8 CHD patients and 8 healthy controls), who were randomly selected from the total enrolled population of 624 participants. The training cohort for model development comprised 422 participants (211 CHD, 211 controls), and the validation cohort comprised 202 participants (101 CHD, 101 controls). Participants were recruited at the Department of Cardiology, Shenzhen Central Hospital. Shenzhen Longhua District Central Hospital is an affiliated hospital of the Shenzhen Central Hospital Medical Group.

The experimental group comprised 311 CHD patients (191 males, 120 females) admitted to the Department of Cardiology, Shenzhen Central Hospital, from May 2022 to May 2024, with a mean age of 65.61 ± 7.62 years and a mean body mass index (BMI) of 22.17 ± 2.42 kg/m^2^. The control group consisted of 311 healthy volunteers (181 males, 130 females) from the same hospital, with a mean age of 65.57 ± 7.81 years and a mean BMI of 22.23 ± 2.38 kg/m^2^. There were no statistically significant differences in baseline data between the two groups (*p* > 0.05). The training cohort (*n* = 422) and validation cohort (*n* = 202) were randomly split from the 624 participants at a ratio of approximately 2:1, stratified by CHD status. CHD was defined as coronary artery stenosis ≥ 50% on coronary angiography, confirmed by two independent cardiologists. Clinical characteristics included typical angina symptoms, electrocardiographic changes, and elevated cardiac biomarkers as appropriate. The study protocol was approved by the Medical Ethics Committee of Shenzhen Central Hospital (Ethics Approval No.: [2025-026-02]), and all participants provided written informed consent. Complete and traceable clinical data were available for all participants.

Inclusion criteria: (1) No significant abnormalities on coronary angiography (coronary artery lumen stenosis < 10%) or CHD excluded by comprehensive evaluation including electrocardiography, echocardiography, and lipid testing; (2) Age and sex matched 1:1 with the CHD group, age range 40–80 years; (3) No history of cardiovascular disease or family history thereof, and no risk factors for cardiovascular disease such as hypertension, diabetes, or hyperlipidemia; (4) Laboratory indicators including liver and kidney function, complete blood count, and coagulation function all within normal ranges; (5) Voluntary participation in the study with signed informed consent.

Exclusion criteria: (1) Concomitant renal insufficiency, defined as serum creatinine level > 2 times the upper limit of normal (i.e., 230 μmol/L), previous renal dialysis treatment, or kidney transplantation; (2) Concomitant hepatic insufficiency, defined as serum transaminase level > 2 times the upper limit of normal (i.e., 80 U/L) or diagnosis of cirrhosis; (3) Pregnant or lactating patients; (4) Patients with advanced cancer, severe infection, or other malignant diseases; (5) Patients with incomplete follow-up information; (6) Patients with a history of thyroid disease or use of antithyroid drugs or thyroid hormones; (7) Patients who had undergone heart transplantation.

### 2.2 Untargeted lipidomics detection

Lipids were extracted from plasma using methanol, acetonitrile, and isopropanol and detected using UHPLC-Q-TOF/MS. Chromatographic separation was performed using an ACQUITY UPLC^®^ HSS T3 column (2.1 × 100 mm, 1.8 μm; Waters, USA) maintained at 45°C. The mobile phase consisted of (A) acetonitrile/water (60:40, v/v) containing 10 mM ammonium formate and 0.1% formic acid, and (B) isopropanol/acetonitrile (90:10, v/v) containing 10 mM ammonium formate and 0.1% formic acid. The gradient program was as follows: 0–2 min, 40% B; 2–4 min, 40–70% B; 4–9 min, 70–100% B; 9–13 min, 100% B; 13–13.1 min, 100–40% B; 13.1–15 min, 40% B. The flow rate was 0.3 mL/min, and the injection volume was 2 μL. Mass spectrometry was performed on a Triple TOF 6600 system (SCIEX, USA) equipped with a DuoSpray™ ion source operating in both positive and negative ion modes. The ion source parameters were: ion source gas 1 (GS1) and gas 2 (GS2) at 50 psi, curtain gas at 35 psi, source temperature at 550°C, and ion spray voltage floating at 5500 V (positive) and -4500 V (negative). Mass calibration was performed using the Calibration Delivery System (CDS) prior to each batch. Internal standards (Avanti Polar Lipids, USA) were added during extraction for normalization. Metabolite annotation was performed at confidence level 2 (putatively annotated compounds) according to the Metabolomics Standards Initiative (MSI) guidelines. One quality control (QC) sample was inserted after every 10 test samples. Any signal (ion intensity of 0) absent in more than 50% of QC samples was deleted. Pearson correlation analysis of the QC samples is shown in Supplementary Figure 1. Hotelling’s T^2^ plot of all samples is shown in Supplementary Figures 2. The relative standard deviation (RSD) of QC samples is shown in Supplementary Figures 3. In both positive and negative ion modes, the retention times and response intensities of the chromatographic peaks in the QC samples showed high overlap. Bioinformatics analysis of the lipidomics data was performed using R software, SIMCA-P14.1, and MetaboAnalyst 5.0.

### 2.3 Lipid subfraction typing

Lipid subfraction concentrations were determined using the vertical auto profile (VAP) method [Farooq et al. (23)]. Plasma lipoproteins were separated according to density gradient. Fractions were sequentially eluted from the bottom of the centrifugal tube via puncture, and the cholesterol content and particle concentration of each lipoprotein fraction (including cholesterol and particle concentrations of 55 analytes such as RLP, VLDL subfractions, IDL subfractions, LDL subfractions and HDL subfractions) were quantified. All lipoprotein subfraction detection kits based on VAP technology and their supporting devices were purchased from Ningbo Medical System Biotechnology Co., Ltd.

### 2.4 Predictive model screening and selection

Least Absolute Shrinkage and Selection Operator (LASSO) regression was applied for feature selection using the glmnet package in R (version 4.2.0). Ten-fold cross-validation was performed to determine the optimal penalty parameter lambda (λ), which was selected as the minimum value of the cross-validated deviance (λ_min). Variables with non-zero coefficients at λ_min were retained for subsequent multivariate logistic regression analysis. Supplementary Figure 4 shows the coefficient paths and cross-validation curves for the LASSO procedure. GraphPad Prism software was used to establish the logistic regression risk prediction model. SPSS 26.0 statistical software was used for data analysis. Quantitative variables were expressed as mean ± standard deviation (x ± s), and comparisons between groups were made using independent samples *t*-tests. Qualitative variables were expressed as frequencies and percentages, and comparisons between groups were made using chi-square tests. All statistical tests were two-sided, and a *p*-value < 0.05 was considered statistically significant. For multiple comparisons in lipidomics data, the false discovery rate (FDR) was controlled using the Benjamini–Hochberg procedure, and an adjusted *P*-value < 0.05 was considered statistically significant for differential lipid identification. All continuous variables were standardized prior to model fitting to facilitate interpretation of regression coefficients. Multivariate logistic regression analysis was used to screen for independent risk factors for CHD. A CHD risk prediction model was constructed based on the identified core lipid markers and clinical indicators. The ROC curve was used to evaluate the discriminative ability of the model, calculating AUC, sensitivity, specificity, and 95% confidence interval (95% CI). Calibration plots, Brier scores, and decision-curve analyses were provided to comprehensively assess model performance.

## 3 Results

### 3.1 Lipidomics detection results

**FIGURE 1:**
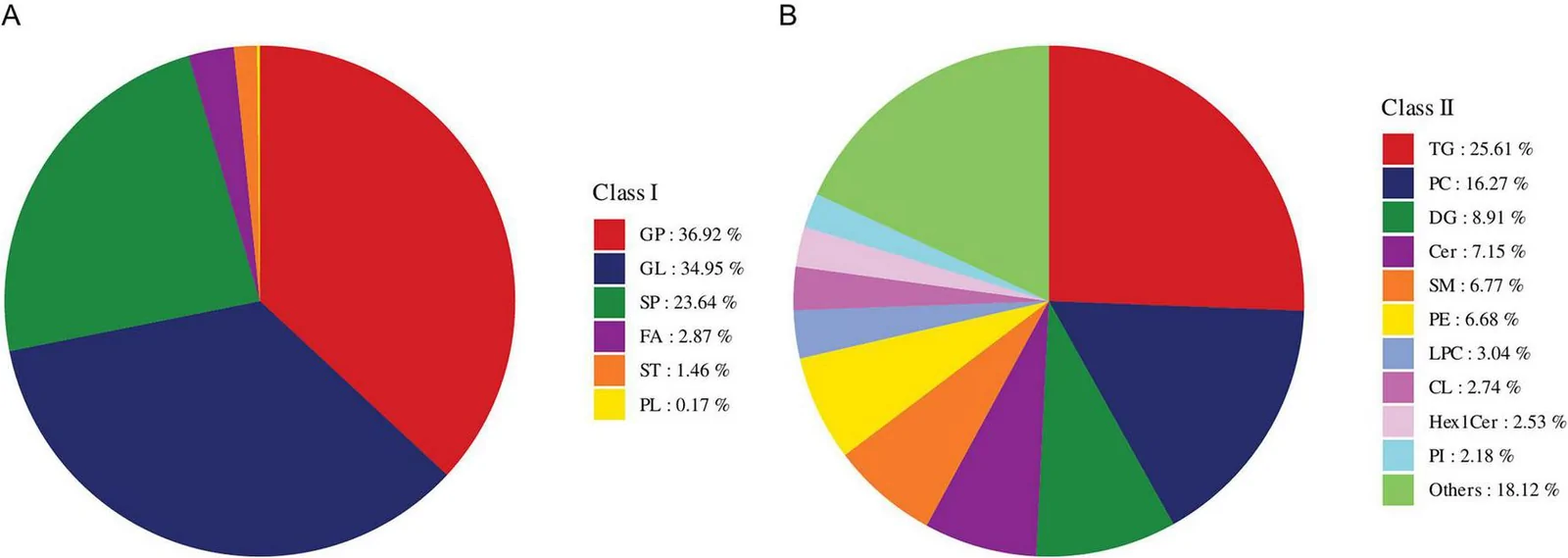
Study design and lipid class composition. Total enrolled participants: 624 (312 CHD, 312 controls). Discovery cohort (lipidomics): *n* = 16 (8 CHD, 8 controls). Training cohort (model development): *n* = 422 (211 CHD, 211 controls). Validation cohort: *n* = 202 (101 CHD, 101 controls). **(A)** Proportion of Class I-identified lipids by chemical class according to the LIPID MAPS^®^ classification system. A total of 2,335 lipid molecules were identified across six classes: glycerophospholipids (GP, 36.92%), glycerolipids (GL, 34.95%), sphingolipids (SP, 23.64%), fatty acids (FA, 2.87%), sterol lipids (ST, 1.46%), and prenol lipids (PL, 0.17%). **(B)** Class II lipid subclass composition, showing the detailed distribution of lipid subclasses within each major class.

Untargeted lipidomics identified a total of 2,335 lipid molecules, classified into 8 categories according to the LIPID MAPS^®^ classification system (Class I): glycerophospholipids (GP, 36.92%), glycerolipids (GL, 34.95%), sphingolipids (SP, 23.64%), fatty acids (FA, 2.87%), sterol lipids (ST, 1.46%), saccharolipids (SL, 0.78%), prenol lipids (PR, 0.15%), and polyketides (PK, 0.13%) (Figure 1A). Figure 1B further illustrates the composition of Class II lipid subclasses, providing a more detailed view of the lipid profile distribution. The sample size for the lipidomics discovery cohort was *n* = 16 (8 CHD, 8 controls), which is suitable for exploratory biomarker screening.

### 3.2 Multivariate statistical analysis of differential metabolites

Table 1 lists the 20 most significantly differential lipid species, including their fold changes, *P*-values, and classification details. In the experimental group, lipid components related to RLP-C and sdLDL-C were primarily characterized by significant upregulation, while HDL2B-related lipids showed a marked downward trend. Triglyceride TG (16:0/16:0/23:0), directly associated with RLP-C, was significantly upregulated (Fold Change = 11.289, *P* = 0.002). Concurrently, glycerolipids related to VLDL (a metabolic precursor of RLP-C) and IDL (an intermediate product), such as TG (36:2e) and TG (12:0e/12:0/12:0), were consistently upregulated, suggesting a close relationship between high glyceride load and remnant lipoprotein accumulation. The sdLDL-C-marked sphingolipid component Cer (t41:1+O) was significantly upregulated (Fold Change = 11.240, *P* = 0.034), and the glycerophospholipid PG (42:0), associated with sdLDL particle remodeling, was also upregulated (Fold Change = 11.838, *P* = 0.038). Lipid components related to HDL2B and functional mature HDL were generally significantly downregulated: sphingomyelin SM (d42:1)-3, diglyceride DG (18:1), and triglyceride TG (4:0/16:1/18:3), associated with HDL2C, HDL2A, and HDL-2 subfractions, were all significantly decreased (*P* < 0.05), suggesting an unfavorable alteration in the lipid composition of HDL2B particles (reduced sphingolipids and glycerolipids), potentially impairing their cholesterol efflux capacity. Furthermore, Lp(a)-characteristically enriched sphingolipid components (e.g., SM (d42:1)-2, Hex1Cer (t32:0)) showed bidirectional differences in this study. While upregulation of Cer (t41:1+O) correlates with the pro-aggregatory properties of Lp(a) particles, the downregulation of some sphingomyelins reflects the complex regulatory patterns of different lipid subfractions in lipoprotein metabolism. After FDR correction using the Benjamini–Hochberg procedure (adjusted *P* < 0.05), the differential lipids remained statistically significant. The lipidomic findings presented here should be considered exploratory and hypothesis-generating, requiring validation in larger independent cohorts.

**TABLE 1:** List of 20 differential lipid class names.

No	Lipid name	Class I	Chinese classification I	Class II	Type	Chinese classification II	Fold change	*P*-value
1	Cer(t41:1+O)	SP	Sphingolipids	Cer	Up	Cer	11.240	0.034
2	DG(38:1)	GL	Glycerolipids	DG	Up	VLDL3	11.357	0.035
3	TG(36:2e)	GL	Glycerolipids	TG	Up	VLDL3a	11.327	0.037
4	PG(42:0)	GP	Glycerophospholipids	PG	Up	sdLDL-C	11.838	0.038
5	TG(12:0e/12:0/12:0)	GL	Glycerolipids	TG	Up	IDL	21.195	0.020
6	TG(18:0/16:0/18:3)	GL	Glycerolipids	TG	Up	IDL1	11.110	0.023
7	TG(18:0/18:0/18:1)	GL	Glycerolipids	TG	Up	IDL2	11.311	0.020
8	TG(16:0/16:0/23:0)	GL	Glycerolipids	TG	Up	RLP-C	11.289	0.002
9	TG(20:3e/20:2/20:2)	GL	Glycerolipids	TG	Up	VLDL3b	11.277	0.025
10	TG(18:3/17:1/18:2)	GL	Glycerolipids	TG	Up	LDL2	10.642	0.018
11	DG(35:2e)	GL	Glycerolipids	DG	Up	LDL3	10.676	0.003
12	DG(16:0/18:2)	GL	Glycerolipids	DG	Up	LDL4	10.928	0.021
13	PC(34:1)	GP	Glycerophospholipids	PC	Down	LDL1	0.001	0.002
14	ST(d37:3+2O)-2	SP	Sphingolipids	ST	Down	Sulf	0.003	0.008
15	SM(d42:1)-3	SP	Sphingolipids	SM	Down	HDL2C	0.002	0.006
16	DG(18:1)	GL	Glycerolipids	DG	Down	HDL-2	0.004	0.004
17	Hex1Cer(t32:0)	SP	Sphingolipids	Hex1Cer	Down	HexCer	0.006	0.012
18	DG(13:0/12:1)	GL	Glycerolipids	DG	Down	HDL-3	0.009	0.008
19	TG(4:0/16:1/18:3)	GL	Glycerolipids	TG	Down	HDL2A	0.013	0.023
20	SM(d42:1)-2	SP	Sphingolipids	SM	Down	SM	0.024	0.031

**FIGURE 2:**
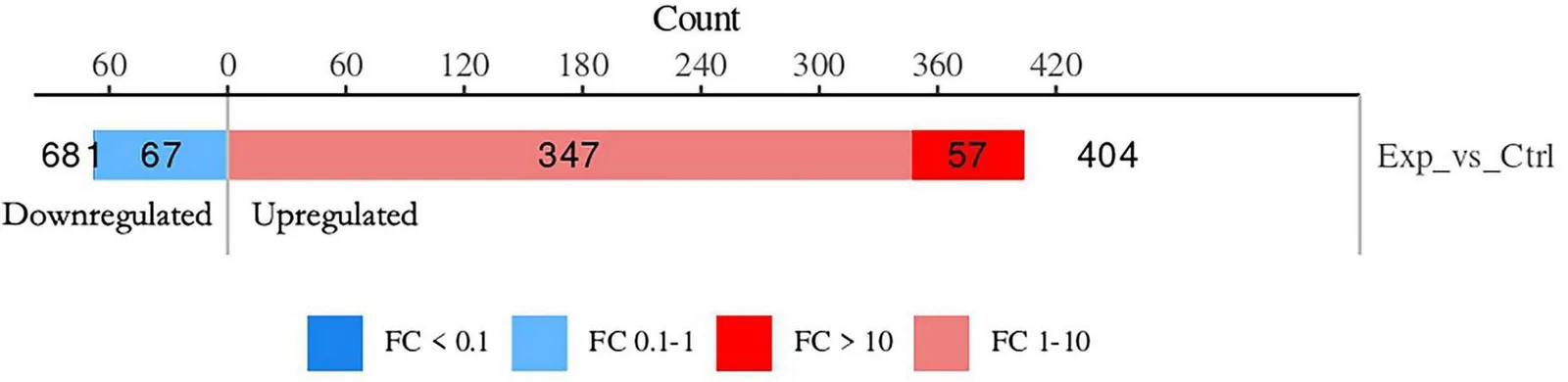
Bar chart of differential lipid counts. The chart summarizes the number of significantly upregulated (red) and downregulated (blue) lipid species across different lipid classes between the CHD and control groups. Statistical significance was determined by nominal *P* < 0.05, followed by false discovery rate (FDR) correction (Benjamini–Hochberg, adjusted *P* < 0.05). Sample size: *n* = 16 (8 CHD, 8 controls).

**FIGURE 3:**
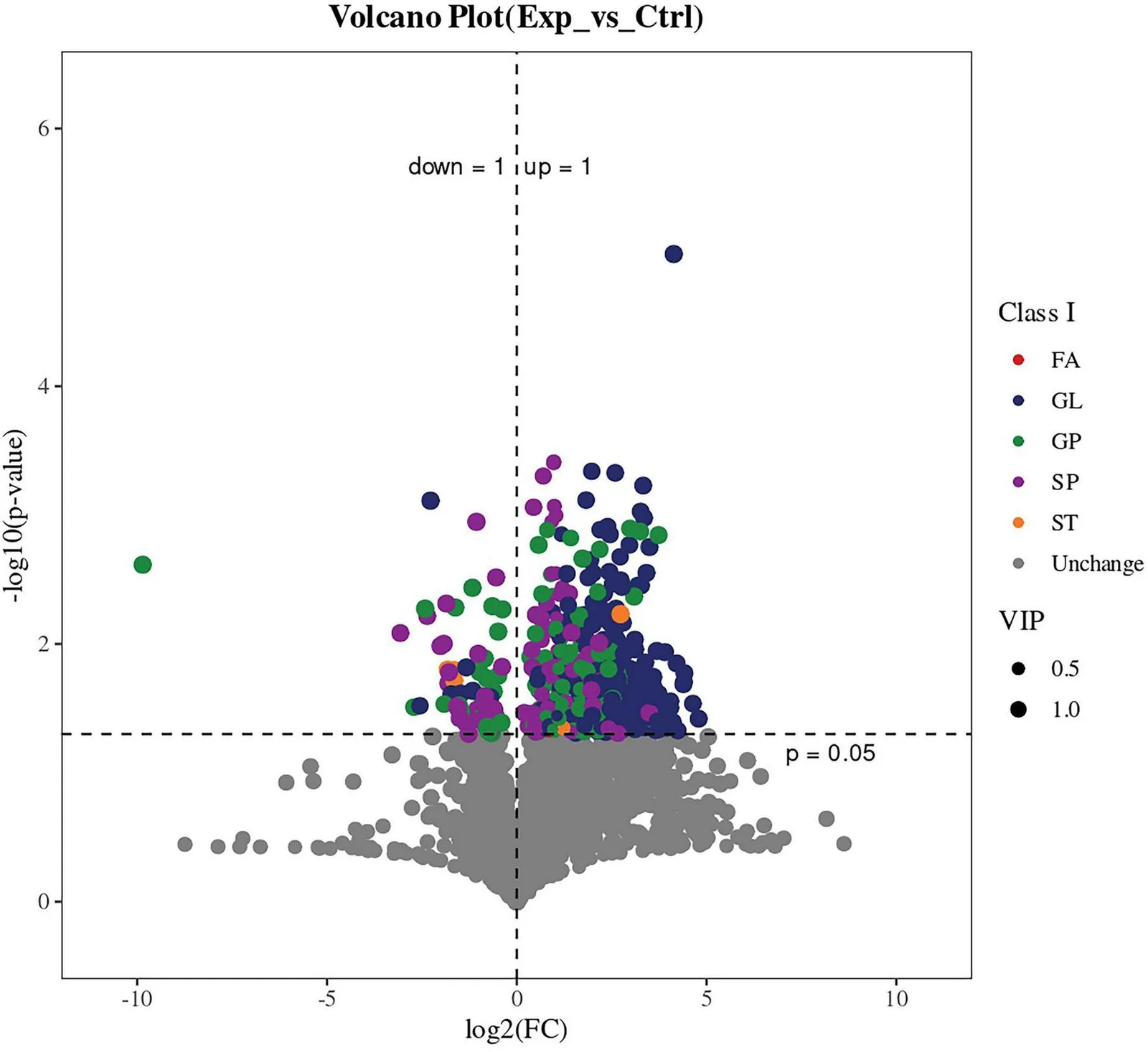
Volcano plot of differential lipid classification. The x-axis represents the log2-transformed fold change (FC) between the CHD and control groups, where positive values indicate upregulation and negative values indicate downregulation in CHD patients. The y-axis represents the -log10-transformed *P*-value, with higher values indicating greater statistical significance. Each dot represents an individual lipid species. Dot size reflects the variable importance in projection (VIP) score derived from partial least squares discriminant analysis (PLS-DA); larger dots indicate greater contribution to group separation. Colors indicate: red = significantly upregulated lipids (adjusted *P* < 0.05, FC > 1.5); blue = significantly downregulated lipids (adjusted *P* < 0.05, FC < 0.67); gray = non-significant changes; yellow = lipids with undefined chemical classification. Statistical significance was determined using the Benjamini–Hochberg FDR correction. Sample size: *n* = 16 (8 CHD, 8 controls). FC, fold change; VIP, variable importance in projection; FDR, false discovery rate.

**FIGURE 4:**
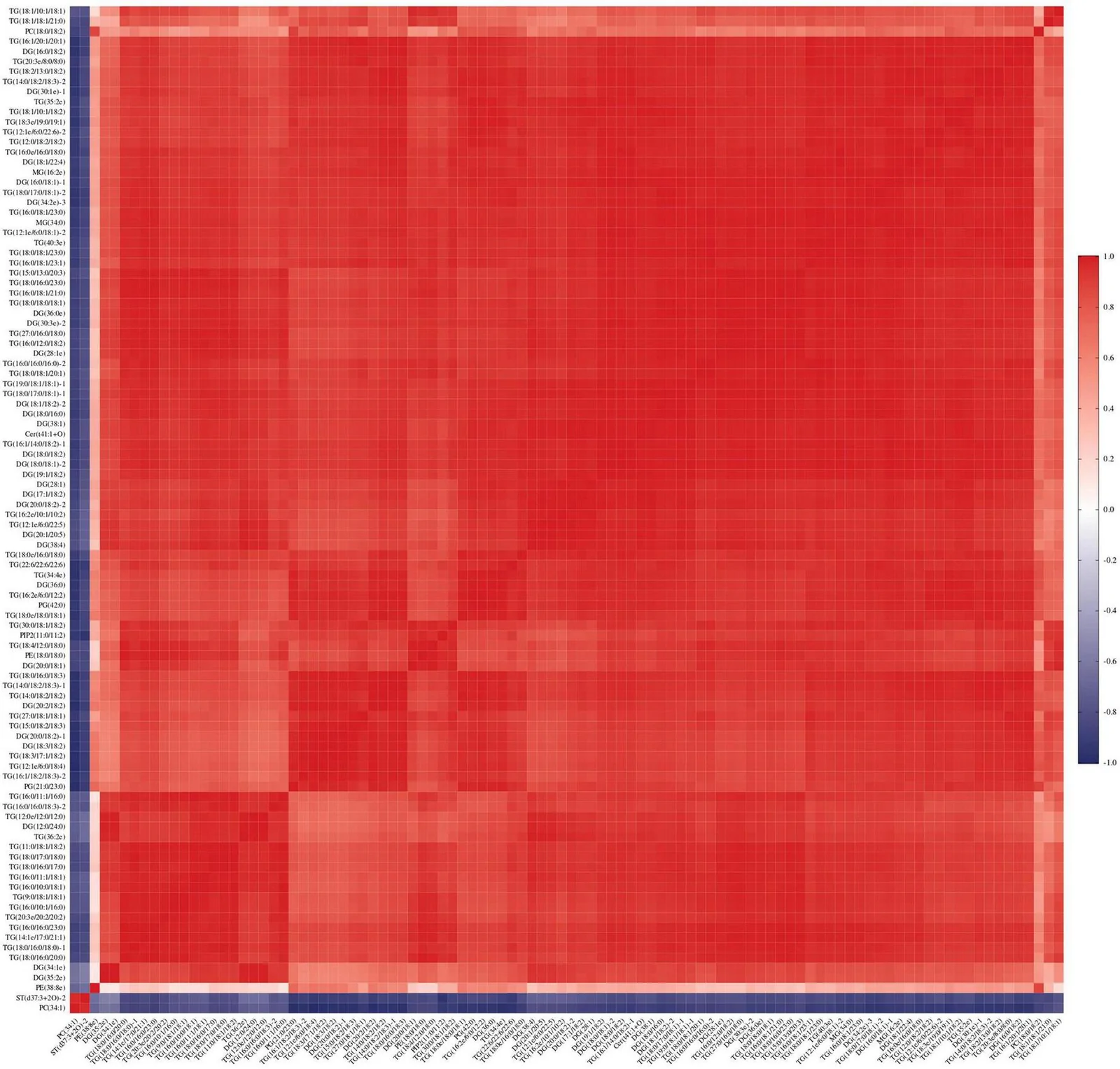
Correlation heatmap of differential lipids. The heatmap displays the Pearson correlation coefficients (r) between all differentially expressed lipid species identified in the study. The color gradient ranges from dark red (strong positive correlation, *r* = +1.0) through white (no correlation, *r* = 0) to dark blue (strong negative correlation, *r* = –1.0). Hierarchical clustering was performed using Euclidean distance and Ward’s linkage method. The dendrograms on the top and left sides indicate clustering of samples and lipid species, respectively. Samples from the experimental group (CHD, *n* = 8) and control group (*n* = 8) show distinct clustering patterns, indicating a CHD-specific lipidomic signature. The heatmap was generated using R software (p heatmap package). Sample size: *n* = 16 (8 CHD, 8 controls). CHD, coronary heart disease; r, Pearson correlation coefficient.

Figure 2 presents a bar chart summarizing the number of upregulated and downregulated lipid species across different classes, highlighting the predominance of glycerolipid and sphingolipid alterations in the experimental group. Figure 3 presents a volcano plot visualizing the differential lipid distribution. The x-axis represents the log2 fold change, the y-axis represents the -log10 *P*-value, and dot size indicates VIP value. Lipids with significant upregulation (red), downregulation (blue), and non-significant changes (gray) are clearly distinguished. In addition, Figure 4 shows a correlation heatmap of the differential lipids, revealing distinct clustering patterns between the experimental and control groups, further supporting the presence of a CHD-specific lipidomic signature.

### 3.3 KEGG/GO pathway enrichment analysis of differential lipids

**FIGURE 5:**
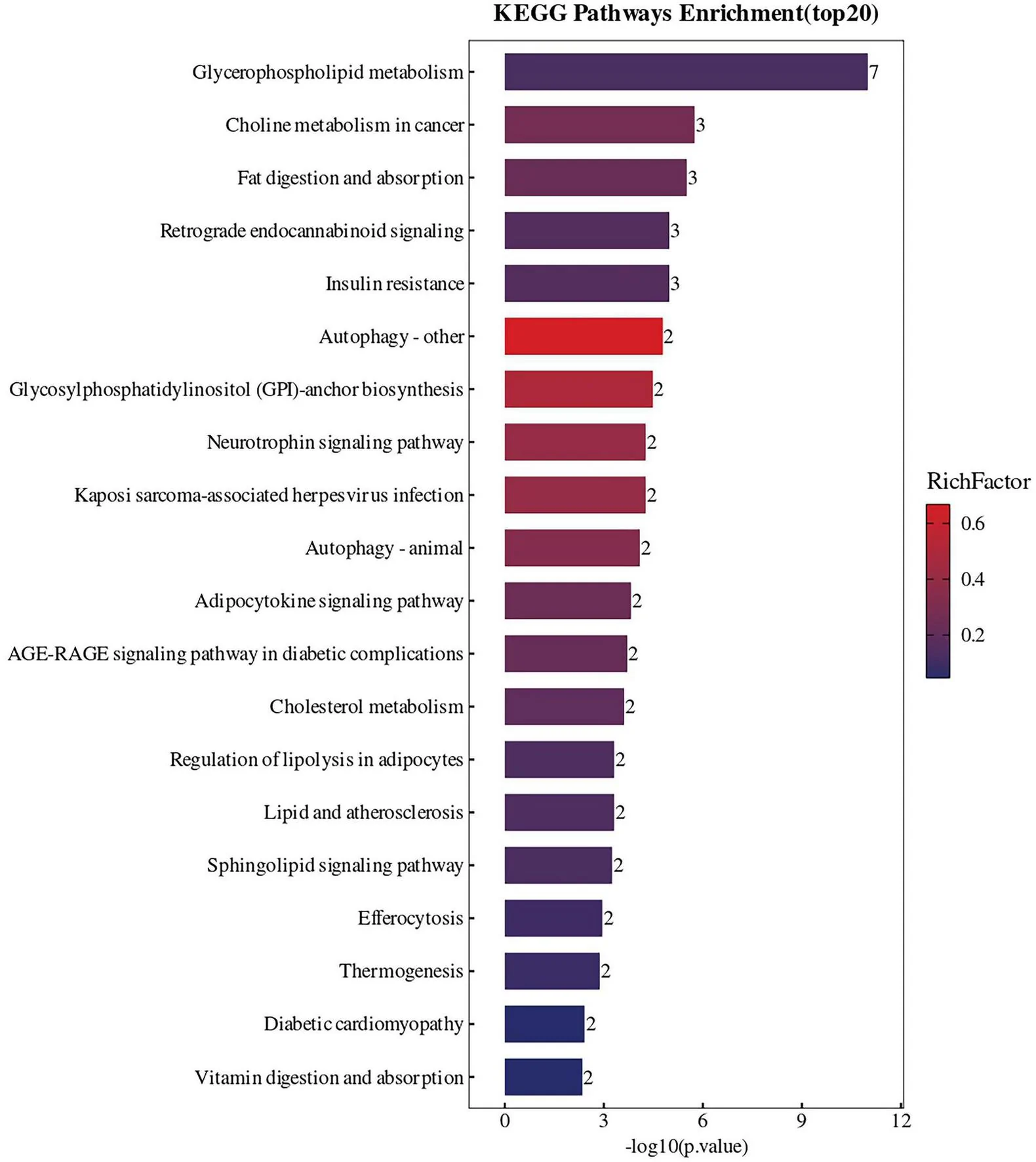
KEGG pathway enrichment analysis of differential lipids. The bar chart shows the top 12 significantly enriched Kyoto Encyclopedia of Genes and Genomes (KEGG) metabolic pathways among the differential lipids (adjusted *P* < 0.05, Benjamini–Hochberg FDR correction). The *x*-axis represents the -log10-transformed adjusted *P*-value, and the *y*-axis lists the pathway names. The top 3 significantly enriched pathways were: (1) Glycerophospholipid metabolism (adjusted *P* = 0.003), (2) Choline metabolism in cancer (adjusted *P* = 0.005), and (3) Fat digestion and absorption (adjusted *P* = 0.008). In the glycerophospholipid metabolism pathway, upregulated differential lipids mainly included phosphatidylcholines (PC) and phosphatidylethanolamines (PE), while downregulated differential lipids mainly included phosphatidylglycerols (PG) and phosphatidic acids (PA). Sample size: *n* = 16 (8 CHD, 8 controls). KEGG, Kyoto Encyclopedia of Genes and Genomes; FDR, false discovery rate; CHD, coronary heart disease.

KEGG pathway enrichment analysis showed that differential lipids were mainly enriched in 12 metabolic pathways. The top 3 significantly enriched pathways were Glycerophospholipid metabolism, Choline metabolism in cancer, and Fat digestion and absorption (*P* < 0.01) (Figure 5). In the glycerophospholipid metabolism pathway, upregulated differential lipids mainly included PC and PE, while downregulated differential lipids mainly included PG and PA, suggesting that disordered glycerophospholipid metabolism may be a core feature of abnormal lipid metabolism in CHD.

**FIGURE 6:**
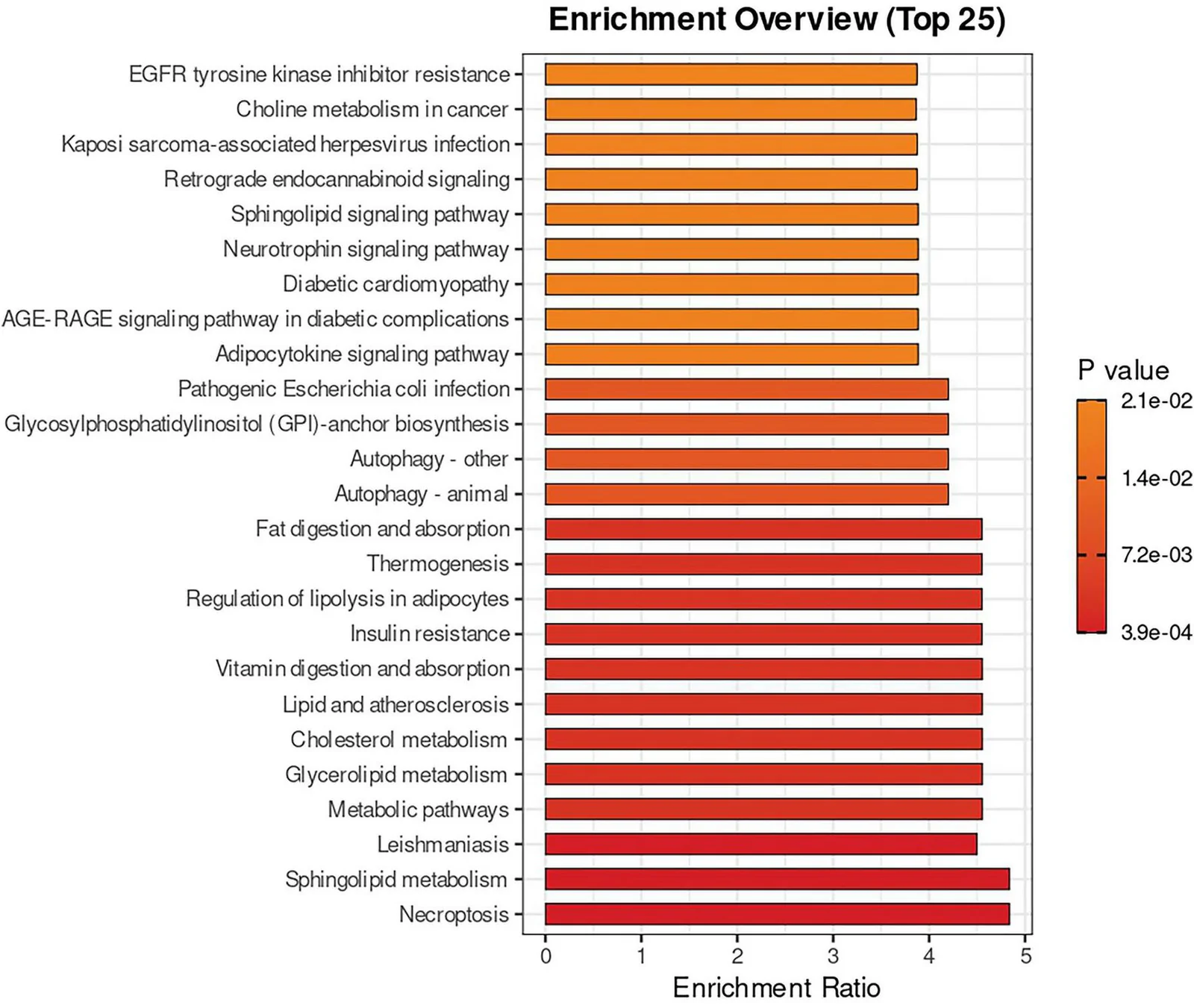
MSEA enrichment analysis plot. Metabolite Set Enrichment Analysis (MSEA) showing the top 25 significantly enriched metabolite sets among the differential lipids. The x-axis represents the -log10-transformed *P*-value, and the y-axis lists the metabolite set names. The size of each dot reflects the number of differential lipids mapped to that set, and the color intensity represents the significance level (darker red = higher significance). Sets related to cardiovascular disease—including the Sphingolipid signaling pathway (adjusted *P* = 0.002), Neurotrophin signaling pathway (adjusted *P* = 0.004), and Diabetic cardiomyopathy (adjusted *P* = 0.006)—were among the top enriched pathways, confirming that the metabolic pathways involving differential lipids are closely associated with the development and progression of CHD. Statistical significance was determined using the Benjamini–Hochberg FDR correction. Sample size: *n* = 16 (8 CHD, 8 controls). MSEA, Metabolite Set Enrichment Analysis; FDR, false discovery rate; CHD, coronary heart disease.

MSEA analysis showed that differential lipids were significantly enriched in 25 metabolite sets, including pathways related to cardiovascular disease such as the Sphingolipid signaling pathway, Neurotrophin signaling pathway, and Diabetic cardiomyopathy (Figure 6), further confirming that the metabolic pathways involving differential lipids are closely associated with the development and progression of CHD.

### 3.4 Characteristics of lipid subfraction levels in CHD

**FIGURE 7:**
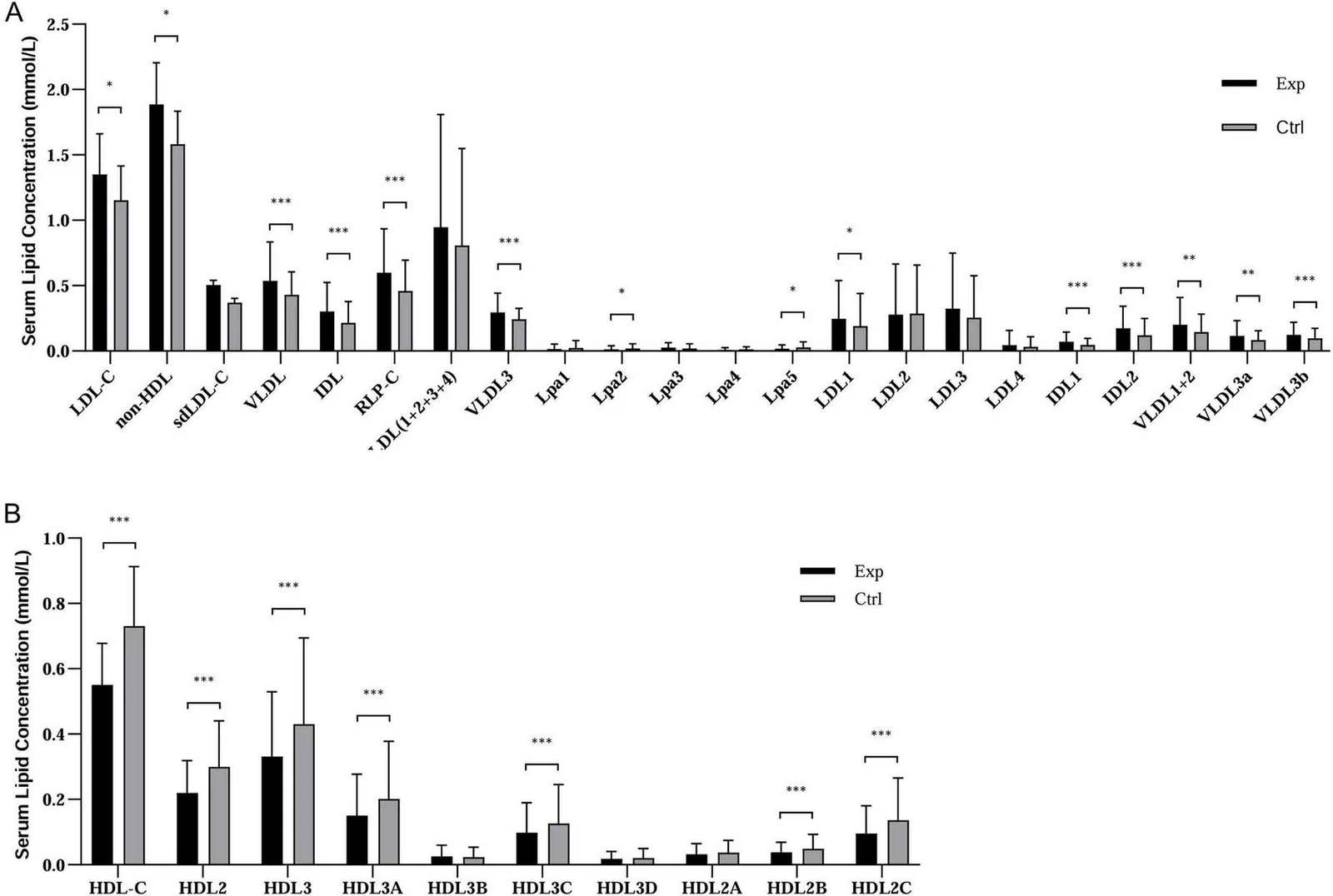
Lipid subfraction levels in CHD patients. **(A)** Comparison of pro-atherogenic lipid subfractions between the CHD group (*n* = 312) and control group (*n* = 312). Data are presented as mean ± standard error of the mean (SEM). Statistical significance was determined by independent samples *t*-test: *P < 0.05, **P < 0.01, ***P < 0.001. The CHD group exhibited significantly higher levels of LDL-C, non-HDL-C, sdLDL-C, VLDL, IDL, RLP-C, VLDL3, total LDL subfractions, LDL1, IDL1, IDL2, VLDL1+2, VLDL3a, and VLDL3b. Among Lp(a) subfractions, only Lpa2 and Lpa5 showed significant increases. **(B)** Comparison of HDL and its subfractions between the CHD and control groups. HDL-C, HDL2, HDL3, HDL3A, HDL3C, HDL2B, and HDL2C were all significantly decreased in the CHD group, with highly significant differences (*P* < 0.001) for HDL-C, HDL3, HDL3A, HDL2B, and HDL2C. Sample size: CHD group, *n* = 312; control group, *n* = 312. CHD, coronary heart disease; LDL-C, low-density lipoprotein cholesterol; non-HDL-C, non-high-density lipoprotein cholesterol; sdLDL-C, small dense low-density lipoprotein cholesterol; VLDL, very low-density lipoprotein; IDL, intermediate-density lipoprotein; RLP-C, remnant-like particle cholesterol; Lp(a), lipoprotein(a); HDL, high-density lipoprotein; SEM, standard error of the mean.

Compared with the control group, the experimental group exhibited significantly higher levels of several pro-atherogenic lipid subfractions (Figure 7). Core lipid indicators LDL-C, non-HDL-C, and sdLDL-C were all significantly increased (*p* < 0.05), with non-HDL-C showing the most prominent increase. Levels of VLDL, IDL, RLP-C, and VLDL3 were also significantly increased (*p* < 0.05–0.001), with IDL showing a highly significant difference (*p* < 0.001). Total LDL subfractions (LDL (1+2+3+4)) and LDL1 were significantly increased (*p* < 0.05). Although LDL2–LDL4 showed increasing trends, they did not reach statistical significance. Among Lp(a) subfractions, only Lpa2 and Lpa5 were significantly increased (*p* < 0.05), while other subfractions showed no significant changes. Additionally, IDL1, IDL2, VLDL1+2, VLDL3a, and VLDL3b were all significantly increased (*p* < 0.05–0.01), indicating that the lipid metabolic disorder in the experimental group was primarily characterized by a comprehensive elevation of triglyceride-rich lipoprotein remnants and small, dense LDL (Figure 7).

Compared with the control group, the levels of high-density lipoprotein (HDL) and its subfractions were significantly reduced in the experimental group: HDL-C (*p* < 0.001), HDL2 (*p* < 0.05), HDL3 (*p* < 0.001), HDL3A (*p* < 0.001), HDL3C (*p* < 0.01), HDL2B (*p* < 0.001), and HDL2C (*p* < 0.001) were all significantly decreased, suggesting a widespread reduction in both total HDL and its subfractions in the experimental group, with highly significant differences for HDL-C, HDL3, HDL3A, HDL2B, and HDL2C (Figure 7).

### 3.5 Construction and validation of the CHD prediction model using lipid subfractions

A multivariate logistic regression model was used to analyze the association between lipid subfractions, clinical indicators, and the outcome event. A total of 422 participants were included in the training set, and the model showed good overall fit. The model results indicated significant associations with the outcome event for multiple variables: Lp(a) (OR = 1.288, 95% CI: 0.09370, 0.4384), RLP-C (OR = 3.848, 95% CI: 0.07071, 2.690), sdLDL-C (OR = 5.317, 95% CI: 0.5472, 2.823), age (OR = 1.053, 95% CI: 0.01453, 0.09062), systolic blood pressure (SBP, OR = 1.075, 95% CI: 0.02922, 0.1186), and fasting plasma glucose (FPG, OR = 3.903, 95% CI: 0.9825, 1.777) were risk factors for the outcome event, while HDL2B (OR = 1.416 × 10^−6^, 95% CI: −20.61, −6.736) was a protective factor. The extremely low odds ratio for HDL2B reflects its strong protective effect and the inverse scaling of the variable in the regression model. Variance inflation factor (VIF) analysis revealed no significant multicollinearity (all VIF < 5). The extremely low odds ratio for HDL2B reflects its strong protective effect and the inverse scaling of the variable in the regression model. Variance inflation factor (VIF) analysis revealed no significant multicollinearity (all VIF < 5). The multivariate logistic regression model equation is: logit(p) = -21.22 + 0.2529⋅Lp(a) + 1.348⋅RLP-C + 1.671⋅sdLDL-C - 13.47⋅HDL2B + 0.05180⋅AGE + 0.07227⋅SBP + 1.362⋅FPG (Table 2).

**TABLE 2:** Parameter estimates and odds ratios from the multiple logistic regression model.

Variable	β coefficient	95% CI for β	Odds ratio (OR)	95% CI for OR
Intercept	−21.22	−27.93 to −15.13	6.064e−10	7.411e−013 to 2.687e−007
Lp(a)	0.2529	0.09370 to 0.4384	1.288	1.098 to 1.550
RLP-C	1.348	0.07071 to 2.690	3.848	1.073 to 14.73
sdLDL-C	1.671	0.5472 to 2.823	5.317	1.728 to 16.83
HDL2B	−13.47	−20.61 to −6.736	0.000001416	1.121e−009 to 0.001188
AGE	0.0518	0.01453 to 0.09062	1.053	1.015 to 1.095
SBP	0.07227	0.02922 to 0.1186	1.075	1.030 to 1.126
FPG	1.362	0.9825 to 1.777	3.903	2.671 to 5.913

**FIGURE 8:**
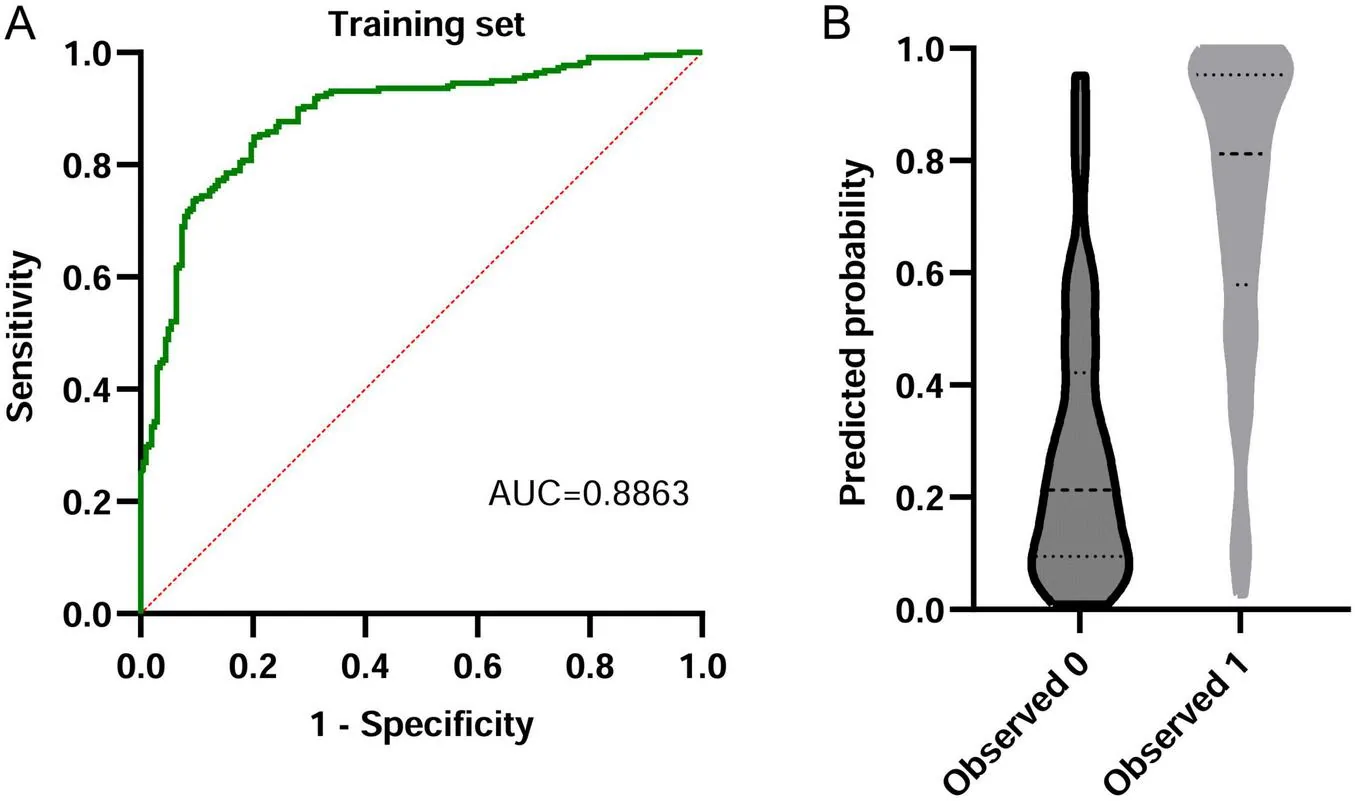
ROC curve and calibration plot of the CHD risk prediction model in the training group. **(A)** Receiver operating characteristic (ROC) curve of the multivariate logistic regression model in the training cohort (*n* = 422, 211 CHD, 211 controls). The area under the curve (AUC) was 0.8863 (95% CI: 0.8541–0.9185, *P* < 0.0001), indicating good discriminative ability. The red dashed line represents the reference line (AUC = 0.5, no discrimination). The optimal cutoff value was 0.5 (Youden index = 0.6038), corresponding to a sensitivity of 78% and specificity of 89% (training cohort). **(B)** Calibration plot showing predicted versus observed probabilities from the multiple logistic regression model. The *x*-axis represents the predicted probability of CHD, and the *y*-axis represents the observed proportion of CHD cases. The diagonal dashed line represents perfect calibration (predicted = observed). The solid blue line shows the locally weighted scatterplot smoothing (LOESS) calibration curve. The Hosmer-Lemeshow test yielded *P* = 0.0021, indicating some deviation in model calibration; however, the calibration curve demonstrates acceptable agreement across the majority of risk deciles, with deviation primarily driven by the highest risk group. The Brier score was 0.089, indicating good overall calibration. The optimism-corrected AUC, estimated using 200 bootstrap resamples, was 0.851. Sample size: training cohort, *n* = 422. ROC, receiver operating characteristic; AUC, area under the curve; CI, confidence interval; CHD, coronary heart disease; LOESS, locally weighted scatterplot smoothing.

Model analysis showed a significant reduction in AICc value compared to the intercept-only model (375.7 vs. 586.4), indicating a substantially improved model fit. The AUC of the model’s ROC curve was 0.8863 (95% CI: 0.8541–0.9185, *P* < 0.0001), suggesting good discriminative ability (Figure 8A). Using a cutoff value of 0.5, the overall classification accuracy of the model was 81.28%, with a negative predictive value of 79.81% and a positive predictive value of 82.71%. Tjur’s R^2^ was 0.4551, indicating good explanatory power of the model for the outcome event. The Hosmer-Lemeshow test yielded a *P*-value of 0.0021, indicating some deviation in model calibration. However, the calibration plot (Figure 8B) demonstrated acceptable agreement between predicted and observed probabilities across the majority of risk deciles, with the deviation being primarily driven by the highest risk group. The Brier score was 0.089, indicating good overall calibration. The optimism-corrected AUC, estimated using 200 bootstrap resamples, was 0.851, suggesting that the model’s discriminative performance is robust against overfitting.

**FIGURE 9:**
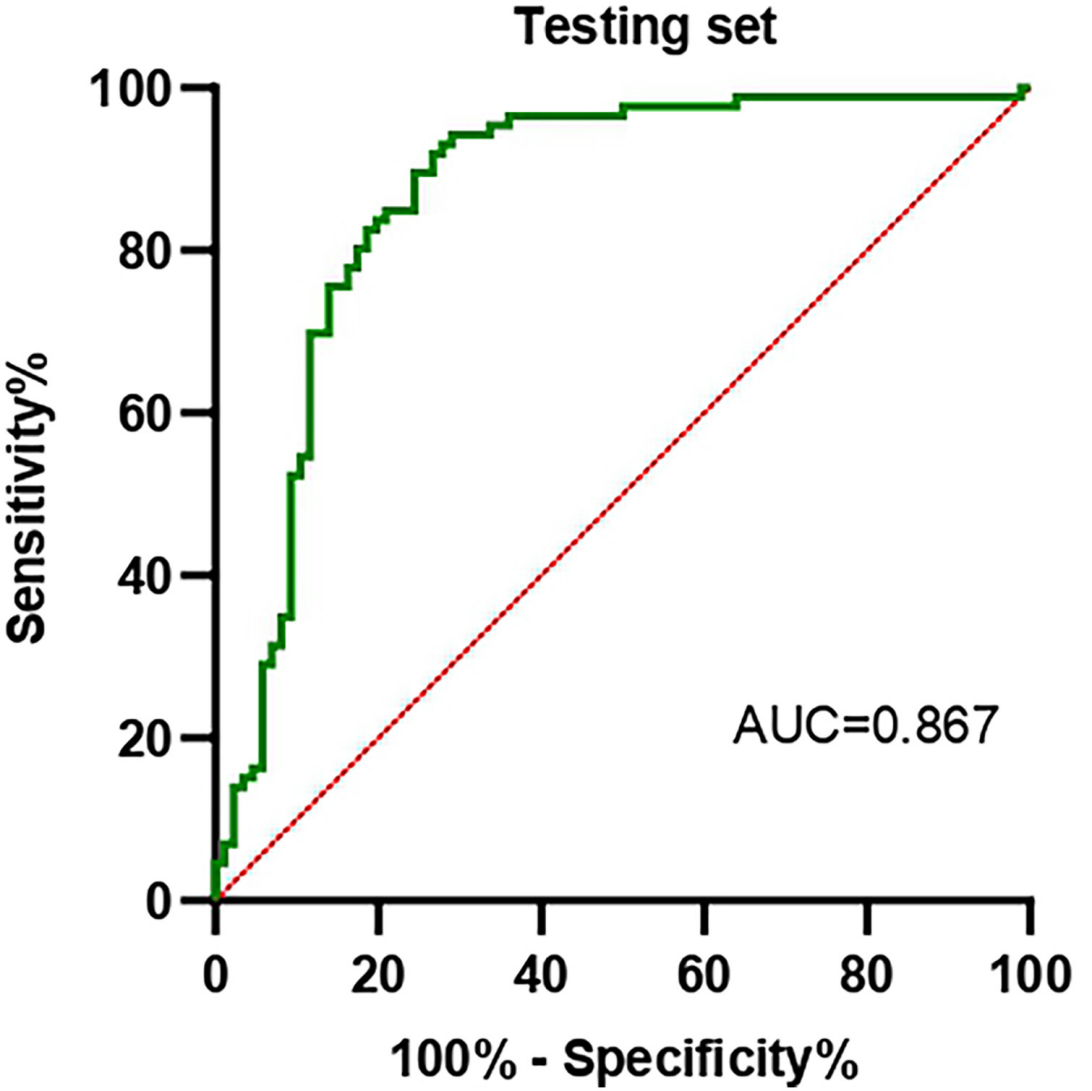
ROC curve of the CHD risk prediction model in the validation group. Receiver operating characteristic (ROC) curve of the multivariate logistic regression model in the internal validation cohort (*n* = 202, 101 CHD, 101 controls). The area under the curve (AUC) was 0.867 (95% CI: 0.8087–0.9252), indicating good discriminative ability. The red dashed line represents the reference line (AUC = 0.5, no discrimination). The optimal cutoff value determined by the maximum Youden index (0.628) was 0.4726, corresponding to a sensitivity of 81.4% (71.89%–88.21%) and a specificity of 81.4% (71.89%–88.21%). The model’s performance in the validation cohort confirms the robustness of the predictive model and supports its potential clinical utility, pending external validation in independent cohorts. Sample size: validation cohort, *n* = 202. ROC, receiver operating characteristic; AUC, area under the curve; CI, confidence interval; CHD, coronary heart disease.

In the validation group (*n* = 202), the AUC of the model was 0.867 (95% CI: 0.8087–0.9252) (Figure 9). The optimal cutoff value determined by the maximum Youden index (0.628) was 0.4726, corresponding to a sensitivity of 81.4% (71.89%–88.21%) and a specificity of 81.4% (71.89%–88.21%).

## 4 Discussion

As the leading cause of death from cardiovascular disease worldwide, the early identification and risk stratification of CHD remain a clinical priority (24). Though conventional lipid indicators are widely used, they still exhibit significant shortcomings in early warning and individualized assessment, primarily due to their inability to capture the functional and structural heterogeneity of lipoproteins (25). Emerging evidence has underscored that conventional lipid measurements miss critical information regarding lipoprotein particle number, size, and composition, which are essential for accurate cardiovascular risk assessment (26, 27). The present study addresses these gaps by integrating untargeted lipidomics with quantitative lipoprotein subfraction analysis (VAP technology) to construct a novel CHD risk prediction model. Our findings demonstrate that a combination of advanced lipid markers, sdLDL-C, RLP-C, Lp(a), and HDL2B, alongside traditional clinical variables (age, SBP, FPG), yields robust discriminative performance, significantly outperforming conventional lipid-based models (28). This integrated approach aligns with the growing consensus that multi-marker lipid phenotyping enhances risk stratification beyond traditional algorithms (29, 30). We acknowledge that the primary novelty of this study lies in the systematic integration of multiple advanced lipid markers including sdLDL-C, RLP-C, HDL2B, and Lp(a) into a single predictive framework, rather than in the discovery of entirely new biological entities. Nevertheless, the combination of lipidomics and VAP-derived subfraction data provides a comprehensive lipid phenotyping approach that may offer incremental value over conventional lipid testing.

After adjusting for traditional risk factors, both sdLDL-C and RC were independent predictors of CHD. Compared with the lowest tertile group, the highest tertile group for combined sdLDL-C+RC had a 2.3-fold increased risk of CHD. Adding these two markers to the traditional risk model significantly increased the C-statistic from 0.702 to 0.765. These findings align with large-scale prospective studies. A recent meta-analysis involving over 500,000 individuals confirmed that elevated sdLDL-C is associated with a 1.5- to 2.0-fold increased risk of CHD, independent of conventional LDL-C levels (31, 32). Using samples from the UK Biobank, researchers analyzed the association between various lipoprotein particle concentrations and subfractions with CHD. The results showed that VLDL, IDL, and LDL particles were positively correlated with CHD, while HDL was negatively correlated. Levels of VLDL, IDL, and RLP-C were significantly elevated in CHD patients, with a stronger risk association for large VLDL particles. Consistent with our findings, it is found that the longitudinal changes in remnant cholesterol are related to the risk of cardiovascular disease (33). In asymptomatic adults without traditional risk factors, serum lipoproteins, particularly LDL-C, ApoB, Lp(a), and low-concentration HDL-C, were independently and strongly correlated with coronary atherosclerosis, reinforcing the concept that traditional risk factors alone are insufficient for comprehensive risk assessment.

The results of this study show that the CHD experimental group exhibited a characteristic dyslipidemic pattern characterized by a widespread increase in pro-atherogenic lipid subfractions and a broad decrease in protective HDL subfractions. Compared with the control group, traditional and novel atherogenic lipid indicators such as LDL-C, non-HDL-C, and sdLDL-C were significantly elevated in the experimental group, with non-HDL-C showing a more pronounced increase. At the same time, levels of triglyceride-rich lipoproteins and their subfractions, including VLDL, IDL, RLP-C, and VLDL3, were significantly increased, with IDL showing a highly significant difference, suggesting that remnant lipoprotein accumulation is a key feature of abnormal lipid metabolism in CHD. This observation is supported by recent genetic studies demonstrating that lifelong elevation of remnant cholesterol due to APOA5 mutations is causally associated with increased CHD risk (34). This finding is clinically important because remnants are highly atherogenic: they are smaller than VLDL but larger than LDL, penetrate the arterial intima easily, and are retained longer due to their high affinity for proteoglycans. Total LDL subfractions and LDL1 were significantly elevated, and LDL2–LDL4 also showed increasing trends. Combined with the upregulation of sdLDL-C, this reflects abnormal LDL particle distribution and an increase in small, dense LDL in CHD patients, which is more prone to subendothelial deposition and pro-atherosclerotic injury due to its reduced affinity for the LDL receptor and increased susceptibility to oxidative modification. Atherosclerosis regression studies have shown that reducing sdLDL-C is associated with plaque stabilization, highlighting its therapeutic relevance (35). For Lp(a) subfractions, only Lpa2 and Lpa5 were specifically increased, suggesting heterogeneous changes among different Lp(a) subfractions in the pathological process of CHD. This heterogeneity may reflect differential glycosylation patterns or apo(a) isoform sizes, which modulate the pro-thrombotic and pro-inflammatory potential of Lp(a). Recent clinical trials targeting Lp(a) have underscored the importance of isoform-specific risk assessment, as individuals with small apo(a) isoforms derive greater benefit from Lp(a)-lowering therapies (36, 37). Meanwhile, HDL-C and HDL2, HDL3 subfractions were significantly downregulated in the CHD group, with particularly prominent and highly significant decreases in HDL2B, HDL2C, and HDL3A. This indicates that CHD patients not only have reduced overall HDL levels but also widespread impairment in HDL subfraction structure and metabolic function, resulting in significantly weakened cholesterol efflux and vascular protective capacity. Given that HDL2B is considered the most efficient subfraction in reverse cholesterol transport, its profound reduction represents a critical functional deficit. Prospective studies have confirmed that HDL2B cholesterol concentration is inversely associated with incident CHD independent of HDL-C, suggesting it may serve as a superior biomarker of HDL function (38, 39). Therefore, the elevation of pro-atherogenic subfractions such as triglyceride-rich lipoprotein remnants and sdLDL-C, combined with the general reduction of HDL and its functional subfractions, represents a characteristic dyslipidemic phenotype of CHD that collectively participates in the occurrence and progression of the disease.

In the experimental group, RLP-C and sdLDL-C-related lipid components were significantly upregulated, while HDL2B-associated protective lipids were markedly downregulated, presenting a pro-atherogenic dyslipidemia phenotype. RLP-C and its metabolic precursors (VLDL, IDL)-related glycerolipids were concurrently elevated, suggesting that high glyceride load drives remnant lipoprotein accumulation, contributing to the pathological process of CHD. Mechanistically, when triglyceride-rich lipoproteins are hydrolyzed by lipoprotein lipase, the resulting remnants are not efficiently cleared by hepatic receptors, leading to their prolonged circulation and arterial infiltration. The upregulation of sdLDL-C characteristic sphingolipids (e.g., Cer(t41:1+O)) and glycerophospholipids (e.g., PG(42:0)) reflects abnormal lipid remodeling that promotes the formation of small, dense LDL. This remodeling process involves cholesteryl ester transfer protein (CETP)-mediated exchange of triglycerides from VLDL into LDL, followed by lipolysis, resulting in smaller, denser particles. Lipidomics studies have identified specific ceramide species as key mediators of LDL trapping and aggregation within the arterial wall, linking sphingolipid metabolism to atherogenesis (40, 41). The general reduction of HDL2B and mature HDL-related sphingomyelins and glycerolipids indicates an imbalance in the lipid composition of HDL2B particles (reduced sphingolipids and glycerolipids), weakening cholesterol efflux and vascular protection (42). Notably, HDL-associated sphingolipids are critical for maintaining HDL structure and stability; their depletion may accelerate HDL catabolism and impair its anti-inflammatory functions. Lp(a)-characteristic sphingolipids showed bidirectional differential expression, suggesting complex regulatory effects of its different lipid subfractions in lipoprotein metabolism and CHD pathogenesis. For instance, while certain sphingomyelins were downregulated, the specific ceramide Cer(t41:1+O) was upregulated, which may contribute to Lp(a)’s pro-inflammatory and pro-apoptotic effects on the vascular wall.

Combining Lp(a), RLP-C, sdLDL-C, and HDL2B with age, systolic blood pressure, and fasting plasma glucose for CHD risk prediction may offer superior stability, discrimination, and fit compared to single traditional indicators. As shown in the results of this study, the CHD prediction model constructed based on these indicators demonstrated good clinical performance, with an AUC of 0.8863 in the training cohort, outperforming many previously reported models that rely on conventional lipids alone. sdLDL-C identifies the small, dense, highly atherogenic LDL phenotype. RLP-C specifically reflects remnant lipoprotein accumulation, capturing the risk associated with postprandial hyperlipidemia and insulin resistance. Lp(a) captures innate, genetically determined high risk that is largely independent of lifestyle. HDL2B precisely reflects the true functional level of HDL, particularly its cholesterol efflux capacity. These markers complement the blind spots of conventional lipids from the perspectives of lipoprotein particle structure, remnant burden, and lipid function, covering multidimensional pathogenic pathways including genetics, metabolism, vascular injury, and lipoprotein function, with good complementarity. Machine learning approaches incorporating these advanced lipid markers have consistently demonstrated superior reclassification metrics compared to traditional risk scores, with net reclassification indices exceeding 20% (43, 44). Importantly, Lp(a) levels are genetically determined and remarkably stable over time, while RLP-C, sdLDL-C, and HDL2B are less influenced by short-term lifestyle fluctuations than conventional TG and LDL-C, making them more reliable for longitudinal risk assessment. The prediction model constructed by combining them with age, blood pressure, and fasting glucose demonstrates good reproducibility and clinical applicability. Joint modeling allows for more precise risk stratification, significantly improving model discrimination, fit, and clinical net benefit. Evaluation of the model in the validation group showed an AUC of 0.867, sensitivity of 81.4%, and specificity of 81.4%, indicating good discriminative ability for CHD. Compared with models relying only on traditional lipids, the inclusion of lipid subfractions significantly enhanced model performance, suggesting that refined lipid markers such as sdLDL-C, RLP-C, and VLDL3 can effectively compensate for the shortcomings of conventional lipid indicators.

To contextualize the performance of our model, we compared its AUC (0.886 in training, 0.867 in validation) with that of the PROCAM model (AUC = 0.824) reported in the literature. However, direct head-to-head comparison with established risk scores such as Framingham, PROCAM, and SCORE2 was not feasible in our dataset due to the unavailability of all required variables and the cross-sectional nature of the study. Future prospective studies should incorporate these comparisons using net reclassification improvement (NRI) and integrated discrimination improvement (IDI) metrics.

The biological plausibility of our findings is strongly supported by existing mechanistic studies. Small, dense low-density lipoprotein cholesterol (sdLDL-C) and remnant-like particle cholesterol (RLP-C) are considered key pro-atherogenic particles, which more readily enter the subendothelial space and induce inflammatory responses (45, 46). Once retained in the intima, these particles undergo oxidation and aggregation, triggering macrophage recruitment, foam cell formation, and subsequent plaque development. VLDL3, as a triglyceride-rich lipoprotein subtype, is closely associated with endothelial injury and plaque progression (47). Its large size and high triglyceride content make it particularly effective at activating inflammatory pathways, including NF-κB-mediated cytokine production. Conversely, HDL3, a subfraction with strong antioxidant and anti-inflammatory functions, has reduced levels indicating diminished protective lipid function (48). HDL3 particles contain high amounts of paraoxonase and platelet-activating factor acetylhydrolase, enzymes that degrade oxidized phospholipids and prevent LDL modification. The reduction in HDL3 thus not only impairs cholesterol efflux but also removes a key line of defense against oxidative vascular injury. In this study, integrating the aforementioned lipids with traditional risk factors resulted in a model that is both stable and practical, potentially providing an objective basis for screening high-risk clinical populations.

It is worth noting that our lipidomics analysis provided molecular-level insights that complement the subfraction data. For example, the significant upregulation of specific triglyceride species such as TG(16:0/16:0/23:0) and TG(12:0e/12:0/12:0) in the experimental group points to specific fatty acid chain compositions that may confer resistance to hydrolysis, thereby prolonging the circulation of remnant particles. Similarly, the downregulation of multiple HDL-associated sphingomyelins (e.g., SM(d42:1)-3) suggests a loss of HDL structural integrity. These molecular signatures could serve as even more precise biomarkers in future targeted assays.

This study has several limitations. First, the discovery cohort for untargeted lipidomics comprised only 16 participants (8 CHD, 8 controls), which limits statistical power and the generalizability of the lipidomic findings. These findings should therefore be considered exploratory and hypothesis-generating, requiring validation in larger independent cohorts through targeted lipidomics. Second, this is a single-center, cross-sectional case–control study. Because participants already had established CHD at enrollment, the model primarily distinguishes existing CHD cases from controls rather than predicting future disease occurrence. Long-term prospective follow-up studies are needed to assess whether these markers predict incident CHD events. Third, although a validation cohort was employed, it was derived from the same institution and population as the training cohort through random splitting. The model therefore lacks true external validation. Independent multi-center validation is necessary before clinical implementation. Fourth, no information was collected regarding statin use, lipid-lowering therapy, glucose-lowering medications, or other cardiovascular treatments. These therapies could substantially influence lipid subfraction profiles and should be incorporated into future studies. Fifth, the Hosmer-Lemeshow test indicated some deviation in model calibration, suggesting that the model’s probability estimates should be interpreted with caution. Sixth, the model did not include genetic polymorphisms (e.g., PCSK9, APOE, LDLR variants), circulating inflammatory markers (e.g., high-sensitivity CRP, IL-6, TNF-α), or advanced imaging parameters (e.g., coronary artery calcium score, carotid intima-media thickness). Future integration of multi-omics data may further improve predictive performance. Finally, the model’s cutoff value of 0.4726 derived from the Youden index, while statistically optimal, requires external validation before clinical implementation.

In summary, this study developed a multivariate logistic regression risk prediction model based on multiple lipid parameters and clinical indicators. However, due to the cross-sectional design, the model primarily distinguishes existing CHD cases from controls rather than predicting future disease onset. The indicators included in the model including Lp(a), sdLDL-C, FPG, RLP-C, HDL2B, age, and systolic blood pressure are all independent factors associated with CHD presence. The model demonstrates acceptable discriminative performance and may provide a complementary tool for CHD risk assessment, pending external validation in larger prospective cohorts. It may serve as a preliminary objective reference for early CHD screening, individual risk stratification, and precision prevention, but its clinical applicability requires confirmation through independent multicenter studies.

## Data Availability

The original contributions presented in the study are included in the article/Supplementary material, further inquiries can be directed to the corresponding authors.
