# Supplementary material for: Development of a CHD risk prediction model using novel lipid markers based on lipidomics and VAP technology
Source: Front Med (Lausanne). 2026 Aug 26;13:1926464. doi: 10.3389/fmed.2026.1926464 (PMC13553228; doi:10.3389/fmed.2026.1926464)
Supplement: Supplementary file 1 [file Data_Sheet_1.docx]

**Supplementary Figures**

Supplementary Figure 1. Correlation plot of QC samples.

Supplementary Figure 2. Hotelling’s T² plot of all samples.

Supplementary Figure 3. MCC plot of QC samples.

Supplementary Figure 4. Relative Standard Deviation of QC Samples.
