# Supplementary material for: Development of a CHD risk prediction model using novel lipid markers based on lipidomics and VAP technology
Source: Front Med (Lausanne). 2026 Aug 26;13:1926464. doi: 10.3389/fmed.2026.1926464 (PMC13553228; doi:10.3389/fmed.2026.1926464)
Supplement: Supplementary file 2 [file Table_1.docx]

**Lipidomics data：**

BioProject：subPRO099268（https://ngdc.cncb.ac.cn/gsub/submit/bioproject/list）
